# Extracellular Vesicles Secreted by Astroglial Cells Transport Apolipoprotein D to Neurons and Mediate Neuronal Survival Upon Oxidative Stress

**DOI:** 10.3389/fncel.2018.00526

**Published:** 2019-01-10

**Authors:** Raquel Pascua-Maestro, Esperanza González, Concepción Lillo, Maria D. Ganfornina, Juan Manuel Falcón-Pérez, Diego Sanchez

**Affiliations:** ^1^Instituto de Biología y Genética Molecular-Departamento de Bioquímica y Biología Molecular y Fisiología, Universidad de Valladolid-CSIC, Valladolid, Spain; ^2^Exosomes Group, Metabolomics Unit and Platform, CIC bioGUNE, CIBERehd, Technology Park of Bizkaia, Derio, Spain; ^3^Instituto de Neurociencias de Castilla y León, IBSAL, Universidad de Salamanca, Salamanca, Spain; ^4^IKERBASQUE, Basque Foundation for Science, Bilbao, Spain

**Keywords:** ApoD, exosomes, extracellular vesicles, astrocytes, neurons, oxidative stress, neuroprotection, glia-to-neuron communication

## Abstract

Extracellular vesicle (EV)-mediated glia-to-neuron communication has been recognized in a growing number of physiological and pathological situations. They transport complex sets of molecules that can be beneficial or detrimental for the receiving cell. As in other areas of biology, their analysis is revolutionizing the field of neuroscience, since fundamental signaling processes are being re-evaluated, and applications for neurodegenerative disease therapies have emerged. Using human astrocytic and differentiated neuronal cell lines, we demonstrate that a classical neuroprotective protein, Apolipoprotein D (ApoD), expressed by glial cells and known to promote functional integrity and survival of neurons, is exclusively transported by EVs from astrocytes to neurons, where it gets internalized. Indeed, we demonstrate that conditioned media derived from ApoD-knock-out (KO) astrocytes exert only a partial autocrine protection from oxidative stress (OS) challenges, and that EVs are required for ApoD-positive astrocytic cell line derived medium to exert full neuroprotection. When subfractionation of EVs is performed, ApoD is revealed as a very specific marker of the exosome-containing fractions. These discoveries help us reframe our understanding of the neuroprotective role of this lipid binding protein and open up new research avenues to explore the use of systemically administered ApoD-loaded exosomes that can cross the blood-brain barrier to treat neurodegenerative diseases.

## Introduction

Nervous system function relies on a complex set of cell types interacting and communicating among them. Cell contact-dependent interactions underlie cell adhesion processes important for neural circuit construction and plasticity. However, communication can also take place by secretion of signaling molecules and neurotransmitters. In this line, the discovery of extracellular vesicles (EVs) have brought a new format of interactions to an already multifaceted communication network. EVs produced by most cells, including all nervous system cell types (Frühbeis et al., 2013a; Lopez-Verrilli et al., 2013; Basso and Bonetto, 2016; Guitart et al., 2016; Croese and Furlan, 2018), open up a new mechanism of signal transmission (Valadi et al., 2007; Hervera et al., 2018) that is changing our understanding of how glia and neurons communicate (Frühbeis et al., 2013b; Basso and Bonetto, 2016; Krämer-Albers, 2017).

As the lipid, carbohydrate, protein and nucleic acid composition of EVs (Kalra et al., 2012; Keerthikumar et al., 2015; Kim et al., 2015) is cell type and physiological state-specific (György et al., 2011; Müller, 2012), such feature makes them candidate biomarker tools in many human diseases, including neuronal disorders (Müller, 2012; Cheow et al., 2016). Furthermore, the potential therapeutic use of EVs is particularly important in brain illnesses, given that they can cross the blood-brain barrier (Alvarez-Erviti et al., 2011; Ridder et al., 2014; Krämer-Albers, 2017). In this regard, EVs purposely loaded with neuroprotective molecules are a promising therapy for neurodegenerative disorders (Pandya et al., 2013; Spencer et al., 2014; Rufino-Ramos et al., 2017).

The Lipocalin Apolipoprotein D (ApoD) is mostly expressed in the nervous system and upregulated in response to oxidative stress (OS; Ganfornina et al., 2008; Bhatia et al., 2012, 2013), a challenge that accompanies physiological aging and disease (Ganfornina et al., 2008; Perdomo and Henry Dong, 2009; Bhatia et al., 2012, 2013). Not surprisingly, ApoD is one of the few genes consistently over-expressed in the aging brain of all vertebrate species tested so far (Loerch et al., 2008). Moreover, ApoD expression is boosted in an amazingly wide array of neurodegenerative and psychiatric diseases of diverse etiology (Suresh et al., 1998; Reindl et al., 2001; reviewed by Dassati et al., 2014). ApoD is actively secreted by astrocytes and myelinating glia, and is uptaken by neurons during neural development and differentiation (Sánchez et al., 2002; Ganfornina et al., 2008, 2010; García-Mateo et al., 2014; Pascua-Maestro et al., 2017). At the cellular level, ApoD is found in the endosome-lysosome-autophagosomal compartment (Pascua-Maestro et al., 2017). Such subcellular location is finely regulated, both in glia, that express ApoD, and in neurons that uptake ApoD in a paracrine fashion (Pascua-Maestro et al., 2017). Within the lysosome, ApoD is able to prevent and revert OS-triggered membrane permeabilization (Bhatia et al., 2013; Pascua-Maestro et al., 2017). ApoD has been demonstrated to reduce free radical-generating lipid hydroperoxides to inert lipid hydroxides (Bhatia et al., 2012), a biochemical property that enables the protection and repair of OS-damaged membranes, finally resulting in cell survival. Previous work demonstrate that ApoD pro-survival mechanism is mediated specifically by the control of lipid peroxidation levels both in cultured cells and *in vivo* animal models (Ganfornina et al., 2008; Navarro et al., 2010; Bajo-Grañeras et al., 2011).

ApoD is considered to be secreted through a canonical secretion pathway, and is found associated to serum LDL/HDL (Perdomo and Henry Dong, 2009; Dassati et al., 2014). However, proteomic analyses have identified this Lipocalin in EVs from serum and cerebrospinal fluid (Cheow et al., 2016; Przybycien-Szymanska et al., 2016). Thus, we set up to test whether ApoD is present in glial-derived EVs and contributes, in this cell-cell communication format, to improve neuronal viability and function.

## Materials and Methods

### Animals

ApoD-knock-out (KO) mice were generated by homologous recombination (Ganfornina et al., 2008), and maintained in positive pressure-ventilated racks at 25 ± 1°C with 12 h light/dark cycle, fed *ad libitum* with standard rodent pellet diet (Global Diet 2014; Harlan Inc., Indianapolis, IN, USA), and allowed free access to filtered and UV-irradiated water. To avoid potential maternal effects of ApoD, and to generate wild-type (WT) and ApoD-KO mice of homogeneous genetic background, the experimental cohorts used in this study are the F1 generation of homozygous crosses of ApoD^−/−^ and ApoD^+/+^ littermates born from heterozygous crosses of an ApoD-KO line backcrossed for over 20 generations into the C57BL/6J background.

Mice experimental procedures were approved by the University of Valladolid Animal Care and Use Committee, following the regulations of the Care and the Use of Mammals in Research (European Commission Directive 86/609/CEE, Spanish Royal Decree 1201/2005). No human subject was involved in this study.

### Cell Culture and Treatments

The human astrocytoma 1321N1 and neuroblastoma SH-SY5Y cell lines were obtained from ECACC (86030402) and ATCC (CRL-2266) respectively. Cells were grown at 37°C in a humidity-saturated atmosphere containing 5% CO_2_. Culture medium was replaced twice a week and cells were subcultured at 90% confluence. Cells were counted with Countess Automated Cell Counter (Invitrogen).

1321N1 cells were cultured in Dulbecco-modified Eagle’s medium (DMEM; Lonza) supplemented with heat-inactivated 5% fetal bovine serum (FBS), 1% L-glutamine, and 1% penicillin/streptomycin/amphotericin B (PSA).

SH-SY5Y cells were cultured in DMEM supplemented with 4.5 g/l glucose, heat-inactivated 10% FBS, 1% L-glutamine, 1% PS and 1% nonessential amino acids (Lonza). To subculture, we used 0.25% Trypsin-EDTA (Gibco Life Technologies). SH-SY5Y differentiation was achieved by culturing cells on collagen-treated plates with 3% FBS and 10 μM retinoic acid (Sigma-Aldrich) for 72 h.

Primary astrocytes from WT and ApoD-KO neonatal (0–1 days old) mice were cultured as described (Bajo-Grañeras et al., 2011). Cerebral cortices were quickly dissected, their meninges removed by rolling on filter paper, and pieces of cortex placed in Earle’s Balanced Salt Solution (EBSS) with 2.4 mg/ml DNAse I and 0.2 mg/ml bovine serum albumin (BSA). The tissue was minced with a surgical blade, centrifuged (200 *g*, 2 min), incubated with 10 mg/ml trypsin for 15 min at 37°C (incubation terminated by 10% FBS addition), mechanically dissociated with a Pasteur pipette, and centrifuged (200 *g*, 5 min). The resulting cells were resuspended in DMEM with 10% FBS, 1% L-glutamine, 1% PSA, plated onto cultured flasks, and incubated at 37°C in 5% CO_2_ with 90%–95% humidity. Culture medium was replaced after 24 h and weekly thereafter. Cells were used for experiments after two subculture steps, when >99% of cells are astrocytes (Bajo-Grañeras et al., 2011).

Exogenous addition of ApoD: human ApoD purified from breast cystic fluid (Ruiz et al., 2013) was added (10 nM) to the cell cultures for 2 h.

Paraquat (PQ) treatment: cells were cultured in phenol red-free DMEM supplemented with 1% L-glutamine, 1% PS, and 0.2% charcoal stripped FBS. This medium without additives was used as our low-serum (LS) control condition. Cells were treated for 1–24 h with PQ (500 μM) prepared in LS medium.

To label and track the whole population of EVs produced by a cell (including exosomes and shedding vesicles) the membranous organelles were labeled with Vybrant-DiI (V22888). To label organelles of the phagocytic-endocytic pathway [endosomes, lysosomes and multivesicular bodies (MVB)] we used Dextran-Alexa 488 (D22910), following the manufacturer’s specifications (Molecular Probes). Cells were incubated with the dye solution for 48 h. After removing the culture medium and performing three 5 min washes with phosphate-buffered saline (PBS), cells were incubated overnight and then subcultured alone or in different co-culture formats (see below).

Cells remained in co-culture during 48 h before performing flow cytometry or fluorescence microscopy analyses to test for transfer of vesicles between astrocytes and neurons. Two types of co-cultures were used: cells were subcultured to experimental plates in a 50:50 proportion (mixed cultures), or separated by a 0.4 μm pore membrane (Transwell plates, Corning Inc.) to avoid direct cell-cell contact.

### Conditioned Media Harvesting for EV Preparations

A standard FBS is used for the culture medium used in the maintenance and amplification of each cell type, and for experiments not directed to EV collection. The experiments described below are performed in EV-free media, where FBS was EV-depleted by ultracentrifugation and added (5%) to phenol red-free DMEM with 25 mM HEPES, 4.5 g/l glucose, 1% L-glutamine and 1% PS.

Conditioned media collection: Cells were cultured in EV-free medium and incubated at 37°C in 5% CO_2_ with 90%–95% humidity for 72 h before the culture medium was collected. A total of 24 culture dishes (20 × 10^6^ cells/dish) per condition were necessary for EV preparations from 1321N1 cells, and 66 culture flasks (8 × 10^6^ cells/flask) per conditions of primary astrocytes. After centrifugation at 1,500× *g* for 30 min at 4°C, the culture supernatant was filtered through a 0.22 μm membrane to obtain a debris-free conditioned medium (CM). The filtered culture medium was immediately frozen at −80°C. Two independent pools of media from 1321N1 cells and two from primary astrocytes per genotype were prepared for further vesicle isolation by differential ultracentrifugation (see below).

### Isolation, Fractionation and Analysis of Astrocyte-Derived EVs

To isolate EVs, the stored debris-free CM samples were centrifuged at 10,000× *g* for 30 min and the supernatant was centrifuged at 100,000× *g* for 75 min. The supernatant of this centrifugation was collected for some experiments as EV-depleted CM. The resulting pellet was washed with an excess of PBS, and centrifuged again at 100,000× *g* for 60 min. The pellet was resuspended in cold PBS and stored at −80°C.

EVs size distribution and concentration were analyzed using a NanoSight LM10 system equipped with a fast video capture and particle-tracking software. Vesicles are visualized by light scattering using a light microscope with a Nanoparticles tracking analysis (NTA) software that tracks Brownian motion of individual vesicles. NTA post-acquisition settings were kept constant for all samples, and each video was analyzed to calculate the median vesicle size and concentration estimates (Dragovic et al., 2011). The starting EV-free culture medium was subject to NTA as control for comparison. We also characterized EVs by Western Blot and Transmission Electron Microscopy (TEM).

Glia-derived EVs (either from the 1321N1 human astrocytic cell line or from mouse primary astrocytes) were fractionated in a continuous 0.25–2 M sucrose density gradient (Taylor, 2015). The EV sample was placed on top of the gradient and centrifuged for 16 h at 210,000× *g*, 4°C, in a SW40 Ti rotor. Fractions (1 ml) were collected from top to bottom by using an autodensity-flow gradient fractionator (Labconco). After mixing by vortex, 20 μl of each fraction were separated for density measures. Subsequently, the rest of the volume was diluted in 20 mM HEPES (pH 7.4), and centrifuged 1 h at 110,000× *g*, 4°C, in a TLA-110 rotor. The pellets were resuspended in PBS and stored at −80°C for subsequent immunoblot analysis. Using the volume set aside, the refractive index of each fraction was measured with a high-resolution refractometer (Abbe 2WAJ, PCE Americans, Inc.). Values are translated into density values by use of equivalence tables.

### Immunoblot Analysis

Cell lysates, cultured media (either directly or concentrated 20× by filter centrifugation with 10 KDa cut-off Centricon YM-10; Millipore), isolated EVs, or EV fractions were analyzed by immunoblot. Denaturing and reducing conditions (0.5% SDS, 25 mM DTT) were used to solubilize proteins prior to electrophoresis in order to detect ApoD. Proteins were transferred to PVDF membranes using standard procedures, and exposed to rabbit serum anti-human ApoD (custom made by Abyntek Biopharma against purified ApoD; Ruiz et al., 2013), goat serum anti-mouse ApoD (Santa Cruz Biotechnology), rabbit serum anti-CD81 (GeneTex), mouse anti-flotillin 1 (Becton Dickinson), or rabbit serum anti-BiP (Sigma), and followed by HRP-conjugated secondary antibodies (Santa Cruz Biotechnology). Membranes were developed with ECL reagents (Millipore) and the signal visualized with a digital camera (VersaDoc; BioRad). The integrated optical density of the immunoreactive protein bands was measured in images taken within the linear range of the camera, avoiding signal saturation.

### Electron Microscopy Methods

Primary astrocytes, destined for pre-embedding immunogold labeling of ApoD, were fixed in 4% formaldehyde and 0.3% glutaraldehyde in 0.1 M PB, pH 7.4, for 30 min at 4°C. Following washes in 0.1 M PB, the cells were blocked with 0.1% cold water fish skin gelatin and permeabilized with Tween-20 (0.5%) in Tris-buffered saline (TBS; 20 mM Tris-HCl, 150 mM NaCl). Samples were incubated with rabbit serum anti-human ApoD antibody diluted in blocking solution. After several washes they were incubated with ultra-small gold-conjugated goat anti-rabbit secondary antibodies Electron Microscopy Sciences (EMS) in PBS. After several washes with PBS, samples were post-fixed in 2% glutaraldehyde in PBS for 20 min, washed, and the ultra-small gold particles were silver-enhanced for 20 min at room temperature with AURION R-Gent SE-EM (Silver Enhancement for Electron Microscopy) following the manufacturer indications. Later, samples were post-fixed with 0.5% OsO_4_ in PBS for 20 min at 4°C, washed with PBS, dehydrated through a graded series of ethanol and embedded in Epoxy EMbed-812 resin EMS. Ultrathin sections were obtained with an Ultracut E ultramicrotome (Reichert/Leica), contrasted with uranyl acetate and lead citrate, and analyzed using a JEOL JEM-1011 HR electron microscope with a CCD Gatan ES1000W camera with iTEM software.

EV preparations destined to cryo-electron microscopy were directly adsorbed onto glow-discharged holey carbon grids (QUANTIFOIL, Germany). Grids were blotted at 95% humidity and rapidly plunged into liquid ethane with the aid of VITROBOT (Maastricht Instruments BV, Netherlands). Vitrified samples were imaged at liquid nitrogen temperature using a JEM-2200FS/CR transmission electron microscope (JEOL, Japan) equipped with a field emission gun and operated at an acceleration voltage of 200 kV.

### Immunocytochemistry

Cells attached to poly-L-lysine (Sigma-Aldrich) treated coverslips were fixed with 4% formaldehyde. Following washes in PBS, cells were blocked and permeabilized with Tween-20 (0.1%) and 1% non-immune (goat or donkey) serum. Cells were incubated with primary antibodies: mouse anti-CD81 (monoclonal JS81), Mouse anti-CD63 (H5C6) or rabbit serum anti-human ApoD. All antibodies were prepared in blocking solution. Cy5, Cy3 (Abcam), Alexa Fluor^®^ 594/488 (Jackson Labs) or DyLight^®^ 405 (Thermo Scientific) conjugated IgGs were used as secondary antibodies. After washes in PBS, cells were mounted in EverBrite™ Mounting Medium with DAPI, and sealed with CoverGrip™ Coverslip Sealant (Biotium).

### Image Acquisition and Analysis

Confocal images were obtained with a 63× oil immersion objective (HCX PL Apo CS NA = 1.4; Leica) attached to a confocal DMI 6000B microscope with a TCS SP5 confocal system (Leica) equipped with AOBS and AOTF systems. Fluorophores were excited with WLL laser (Leica) and a 405 line (Leica) controlled by LAS AF software (Leica). Emissions were collected with the AOBS system and three spectral detectors. Laser power and detection gains were set by scanning control samples labeled with secondary antibody alone. We ensured to obtain similar dynamic ranges in our images, and adjusted gain and offset using LUTs. In this manner, bleed through can be neglected. Negative control images showed very weak and homogeneous background. We obtained confocal sections under constant conditions to minimize image acquisition variation. Images were stored as 1,024 × 1,024 pixels and 8-bit TIFF files.

Z-series (xyz scan) were performed in all cases, covering the whole cell *z*-axis dimension. The number of z-stacks was determined by observing the limits of the cell membrane. The focus plane was set to be 3 μm beneath the section surface. The optimal value of the step size was calculated for the wavelength used to fulfill the Nyquist theorem. The optical section thickness was 0.772 μm. Besides, images were taken with a 4× zoom, reducing field size. Pixel size corresponded to 0.06*0.06*0.3777 μm^3^. Scanning was performed with a 1.0 Airy unit pinhole size.

Images were processed with a Gaussian Blur filter [Sigma (Radius): 1.00], to facilitate object detection, and analyzed with a Colocalization Indices plug-in (Nakamura et al., 2007) and the 3D Object Counter tool of the FIJI software. To analyze triple-colocalization experiments we used the Image Calculator and 3D Object Counter tools of FIJI. We analyzed 20 cells per condition. Colocalization was quantitated using the intensity correlation quotient (ICQ), as described (Li et al., 2004). A 2 × ICQ was adopted so that colocalization values range from −1 (total exclusion) to +1 (total colocalization), with 0.1 representing the threshold for random association of signals (Pascua-Maestro et al., 2017). The index was referenced either to ApoD signal or to DiI signal.

### Flow Cytometry

Cells cultured for 48 h after labeling with Vybrant-DiI or Dextran-Alexa 488 were lifted with 500 μl of Triple (Tryple™ Select, Gibco Life Technologies) after removal of the culture medium and washes with PBS. Suspended cells were analyzed in a FACS Canto II flow cytometer (Beckton Dickinson). DiI signal was collected with the “PE” detector (BP585/42) and Alexa 488 was detected with the “FITC” channel (BP530/30) after excitation with 488 nm laser. Data was processed with Kaluza Analysis software v.1.3 (Beckman Coulter).

### MTT-Viability Assay

Cell viability was measured with the 3-(4,5-dimethylthiazol-2-yl)-2,5-diphenyltetrazolium bromide (MTT) colorimetric assay as previously described (Bajo-Grañeras et al., 2011; Pascua-Maestro et al., 2018). After MTT exposure for 3 h, cells were incubated in isopropanol with 10% Triton X-100, and the solubilized formazan was measured by spectrophotometry using the SOFTmax Pro microplate reader (Molecular Devices). Absorbance was measured at λ = 570 nm after subtracting the λ = 690 nm background.

### RT-PCR

RNAs from 1321N1 cells or differentiated SH-SY5Y cells after exposure to 1321N1-derived EVs were extracted with TRIzol (Qiagen). Total RNA (1 μg) was treated with DNaseI and reverse-transcribed with Prime-Script (Takara). The cDNA obtained was used as template for RT-PCR amplifications. To amplify human ApoD and the housekeeping control gene RPL18 we used the following primers: Human ApoD-Forward: 5’-CCACCCCAGTTAACCTCACA; Human ApoD-Reverse: 5’-CCACTGTTTCTGGAGGGAGA; Human RPL18-Forward: 5’-CCATCATGGGAGTGGACAT-3’; Human RPL18-Reverse: 5’-CACGGCCGTCTTGTTTTC.

### Statistical Analysis

Statistical analyses were performed with SPSS v.19 (IBM) and SigmaPlot v.11.0 (Systat) software. A *p* value < 0.05 was used as a threshold for significant changes. The tests used for each experiment are stated in figure legends.

## Results

### Glia-Neuron Communication *in vitro*

**Figure 1:**
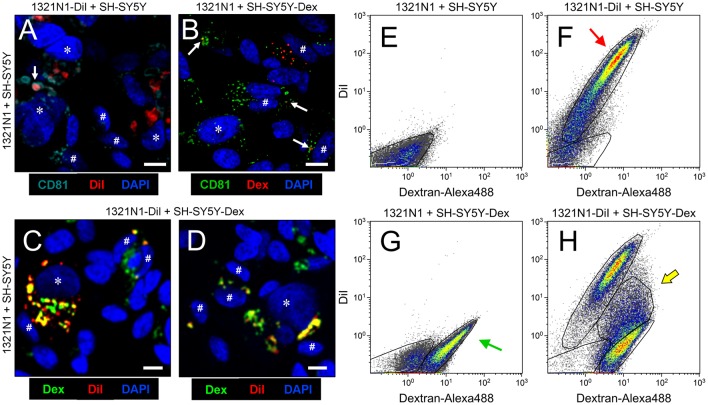
Astrocytes and neurons exchange CD81-positive material. **(A–D)** Single plane confocal microscopy images extracted from Z-stacks obtained from co-cultures of: 1321N1 astrocytes labeled with DiI and unlabeled SHSY5Y neurons **(A)**; unlabeled 1321N1 and SH-SY5Y labeled with Dextran-Alexa 488 **(B)**; 1321N1 labeled with DiI and SH-SY5Y labeled with Dextran-Alexa 488. **(C,D)** Immunocytochemistry with the extracellular vesicle (EV) marker CD81 is shown in **(A,B)**. Arrows point to colocalizing signals. Asterisks and pound signs mark identified nuclei from astrocytes and neurons respectively. *N* = 20 confocal stacks/condition. Calibration bars: 10 μm. **(E–H)** Flow cytometry analyses plotting the DiI and Dextran-Alexa 488 signals in the different co-culture conditions. The red arrow in **(F)** indicates the DiI-1321N1 population, and the green arrow in **(G)** points to the Dextran-Alexa 488-SHSY5Y population. A double labeled cell population appears when both astrocytic and neuronal cells are labeled (**H**, marked with a yellow arrow).

Although extensive data support the role of EVs as mediators of neuron-glia communication *in vivo* (Frühbeis et al., 2013b; Basso and Bonetto, 2016), we tested whether this relationship exists between human astroglial 1321N1 and neuronal SH-SY5Y cells, that have been previously used as a model to unravel the mechanism of action of ApoD (Bajo-Grañeras et al., 2011; Pascua-Maestro et al., 2017). We first studied the possibility of vesicular exchange between these cells by labeling the membranous compartment of 1321N1 astrocytes with the lipophilic compound DiI, and the endo-lysosomal compartment of SH-SY5Y neurons with Dextran-Alexa 488 (see “Material and Methods” section). Co-culturing of labeled cells in EV-free media for 48 h showed colocalization of DiI and Dextran-Alexa 488 in both astrocytes and neurons (Figures 1C,D), suggesting an exchange of membranous material among them. In addition, flow cytometry analysis (Figures 1E–H) confirms the presence of double-labeled cells (DiI-Dextran co-labeling; Figure 1H) and suggests that such transfer of material might be occurring mainly in the astrocyte-to-neuron direction (arrow in Figure 1H). However, the pattern of labeled cells observed might also be due to differences in efficiency of the labeling methods.

**Figure 2:**
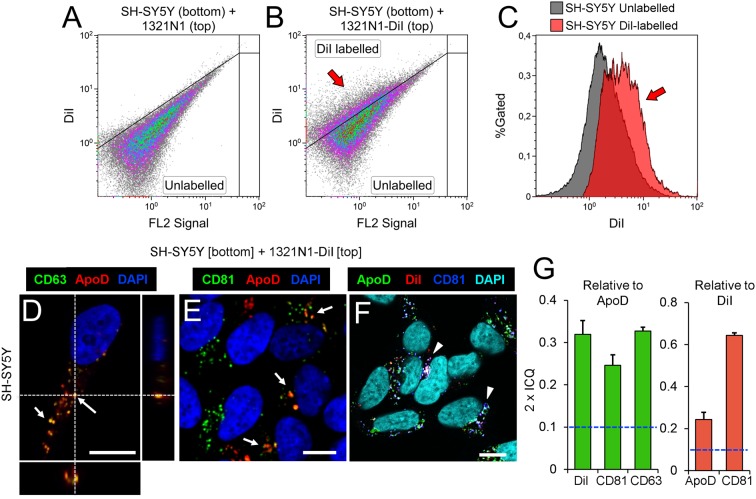
Astrocyte-to-neuron communication is mediated by EVs that carry Apolipoprotein D (ApoD) into neurons. **(A–C)** Flow cytometry analyses of SH-SY5Y cells cultured with 1321N1 cells seeded in a Transwell inset. Astrocytic 1321N1 cells were either unlabeled **(A)** or labeled with DiI. **(B,C)** Dot plots show the existence of a population of DiI-positive SH-SY5Y neurons (arrow in **B**, representing 25.1%) when cultured with labeled 1321N1 astrocytes. The histogram in **(C)** displays the fluorescence increase in DiI-positive neurons (arrow), with median fluorescence increasing from 1.9 to 4.0 (a.u.). **(D–F)** Single plane confocal microscopy images extracted from Z-stacks obtained from unlabeled SH-SY5Y target cells cultured on the lower compartment of a Transwell assay while DiI-labeled 1321N1 donor cells were cultured on the insert. Immunofluorescence with ApoD, CD81 and CD63, and DiI signal are shown with different LUTs to properly display the colocalization of EV markers with ApoD (arrows in **D,E**) or with DiI (arrowheads in F point to triple colocalization DiI-CD81-ApoD). Orthogonal views are shown in **(D)** to visualize the location of vesicular labeling within the cell. *N* = 20 confocal stacks/condition. Calibration bars: 5 μm. **(G)** Average colocalization index (2 × ICQ) referenced either to ApoD signal (green bars) or to DiI signal (red bars) in SH-SY5Y neurons co-cultured in Transwell with DiI-labeled 1321N1 astrocytes. The dotted lines represents the colocalization threshold. Error bars represent SEM (*n* = 20 cells/marker from four independent experiments).

Analyzing DiI and Dextran-Alexa 488 distribution in 1321N1 and SH-SY5Y respectively by confocal microscopy we found that in both cells some of the labeled organelles colocalize with CD81, a marker of late endocytic compartments (Escola et al., 1998; Figures 1A,B). These results suggest that the membranous transfer between cells could be mediated by EVs. Nevertheless, they also could be explained by: (i) a cell-cell direct contact-dependent exchange of material; (ii) endocytosis of cellular debris such as apoptotic bodies generated in the co-cultures; or (iii) endocytosis of EVs produced by either cell type. Thus, to discern between these scenarios we used a Transwell assay, focusing on the transcellular traffic from astrocytes to neurons. Using EV-free media, we cultured DiI-labeled 1321N1 astrocytes as donor cells, on the Transwell insert, and SH-SY5Y neurons as target cells, on the lower compartment. After 48 h of incubation, a DiI positive population of SH-SY5Y recipient cells is detected by flow cytometry (Figures 2A–C). These experiments suggest that SH-SY5Y neurons receive and internalize DiI-labeled EVs secreted by 1321N1 astrocytes.

### Neurons Uptake Glial-Derived EVs Containing ApoD

As ApoD is secreted by astrocytes and uptaken by neurons (Pascua-Maestro et al., 2017), we wanted to test if EVs play a role in such intracellular communication. In contrast to ApoD-expressing glioma and astrocytic cell lines like 1321N1 cells (Bajo-Grañeras et al., 2011; Pascua-Maestro et al., 2017), no ApoD expression has been detected in SH-SY5Y neuroblastoma cells either by genome-wide transcriptome analyses (e.g., 0.0 FPKM for ApoD in http://systemsbiology.uni.lu/shsy5y/) or by immunodetection of ApoD protein (Pascua-Maestro et al., 2017). We therefore tested whether 1321N1-derived vesicles uptaken by SH-SY5Y neurons contain astrocyte-derived ApoD using the Transwell co-culture system.

When co-cultured with 1321N1 cells in the Transwell insert, we are able to detect ApoD signal in intracellular vesicles of SH-SY5Y (Figures 2D–F), with a significant colocalization with the EV markers CD63 (Figures 2D,G) and CD81 (Figures 2E,G), as well as with the astrocyte-originated DiI signal (Figures 2F,G). Although, in general, the detected CD63 and CD81 can be either endogenous (produced by neurons) or transferred in astrocyte-derived EVs, the particular subset of CD63 or CD81-positive organelles that are also ApoD-positive are strong candidates to have an astrocytic origin. Indeed, the high colocalization index (2 × ICQ > 0.6) of CD81 relative to the DiI signal detected in neurons, strongly supports a significant uptake of CD81-DiI-labeled EVs, a fraction of which (2 × ICQ > 0.2), bring ApoD with them (Figure 2G). The significant colocalization indexes agree with the high incidence of triple colocalization of ApoD-CD81-DiI (Figure 2F).

However, ApoD could be provided externally to neurons as protein or mRNA, or alternatively its expression be induced after treatment with astroglial EVs, since the later have been shown to trigger diverse signaling cascades in cells (e.g., Hervera et al., 2018). To distinguish among these three possibilities we cultured SH-SY5Y neurons with: (1) EV-free standard media (SM); (2) 1321N1-conditioned media (CM); (3) the EV fraction (EVs) obtained by differential centrifugation of CM; and (4) the EV-depleted supernatant (Sup) of CM. We then used immunocytochemistry to detect ApoD protein in neurons, and RT-PCR to monitor *APOD* mRNA expression in the donor and recipient cells involved.

**Figure 3:**
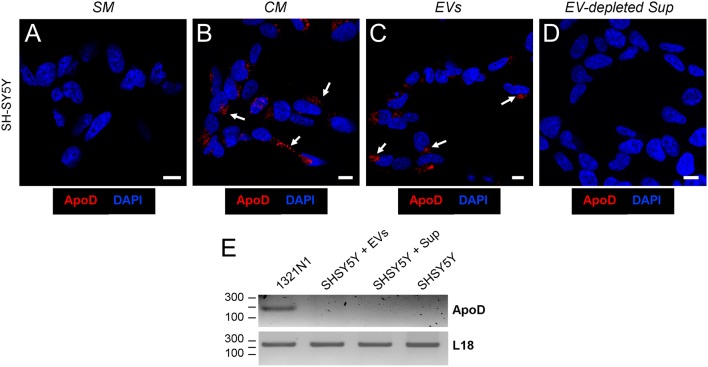
Astrocyte-to-neuron ApoD traffic is exclusively mediated by EVs. **(A–D)** Single plane confocal microscopy images extracted from Z-stacks obtained from SH-SY5Y cells cultured with standard medium (SM; **A**), astrocyte-conditioned medium (CM; **B**), purified astrocyte EVs **(C)**, or EV-depleted astrocyte-derived supernatant, Sup **(D)**. Arrows point to the ApoD vesicular labeling within SH-SY5Y neurons. No signal is detected when SM or EV-depleted Sup is used. Calibration bars: 10 μm. **(E)** RT-PCR analysis of ApoD mRNA expression in the1321N1 donor cells, and in SH-SY5Y differentiated neurons after 48 h exposure to 1321N1-generated EVs. The housekeeping ribosomal gene RPL18 is amplified as control.

Immunofluorescence analysis of cultured SH-SY5Y showed the absence of ApoD in SM-cultured neurons (Figure 3A), thus confirming the absence of endogenous ApoD in control conditions. ApoD protein is observed in CM-cultured neurons (Figure 3B), in agreement with our previous reports showing internalization of exogenously added ApoD in neurons (Pascua-Maestro et al., 2017) and with the results obtained in the Transwell experiments (Figure 2). Surprisingly, when CM is separated into EVs and EV-depleted supernatant, ApoD is only observed in neurons after addition of the fraction containing EVs (Figure 3C) and no ApoD is obtained after exposure to the EV-depleted supernatant (Figure 3D).

Additionally, RT-PCR was performed in order to detect *APOD* mRNA expression in 1321N1 donor astrocytes and SH-SY5Y recipient neurons after 48 h exposure to 1321N1 EVs. While the housekeeping ribosomal gene *RPL18* is present in all samples, *APOD* mRNA was only detected in the donor cells (Figure 3E), thus discarding a potential induction of endogenous *APOD* expression in SH-SY5Y neurons upon exposure to 1321N1-derived EVs followed by *de novo* synthesis of ApoD protein.

### Characterization of ApoD in Glial EVs: ApoD as a Very Specific Marker of Human Astroglial Exosomes

**Figure 4:**
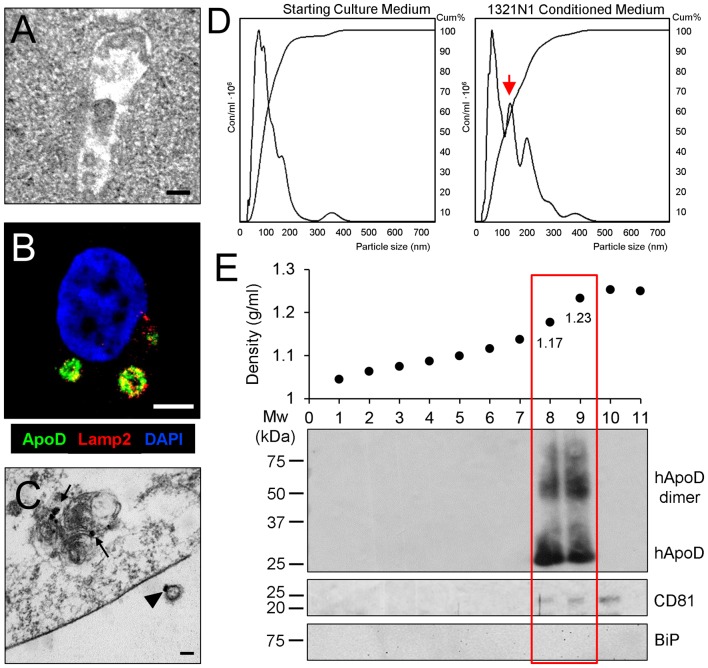
ApoD is specifically found in EV fractions containing astrocyte-derived exosomes. **(A)** Electron microscopy image of a multivesicular body (MVB) in the cytoplasm of a 1321N1 astrocyte. **(B)** Single plane confocal microscopy image extracted from a Z-stack of a 1321N1 astrocyte showing the colocalization of ApoD and Lamp2 in putative MVBs. **(C)** Immunoelectron microscopy image showing ApoD in mature lysosomes of a 1321N1 cell (arrows), and on the external surface of a putative exosome (arrowhead). Calibration bars: **(A,C)**: 100 nm; **(B)**: 5 μm. **(D)** Nanoparticle tracking analysis (NTA) of the starting culture medium or 1321N1-CM shows that 1321N1 astrocytes secrete several EV populations with a group showing particle size compatible with exosomes (red arrow). EV preparations were obtained from two pools of two independent cell culture sets (480 × 10^6^ cells/preparation). Particle size: 92 and 97 nm, respectively. Particle concentration: 218 × 10^9^ and 222 × 10^9^ particles/ml respectively. **(E)** Fractionation of EV preparation from 1321N1 astrocytes by sucrose gradient. The plot shows the fraction density along with the immunoblot analysis of each fraction with ApoD, CD8 and BiP antibodies. Only fractions 8 and 9 are positive for ApoD, while the exosome marker CD81 appears in fractions 8–10.

So far, our results show that 1321N1 astroglia express the EV marker CD81 (Figure 1A), that colocalizes with DiI-labeled membranous organelles and can be transferred to neurons (Figures 2, 3). Also, MVB can be detected in the cytoplasm of 1321N1 astrocytes by EM (Figure 4A). Our previous analysis of intracellular traffic of ApoD (Pascua-Maestro et al., 2017) revealed its presence in the extracellular side of the plasma membrane, and its traffic through the endo-lysosomal and autophagosome compartments. Here, we show by means of immunofluorescence and immunoelectron microscopy that ApoD can also be found both in Lamp2-positive MVBs (Figure 4B), and in putative EVs (arrowhead in Figure 4C), whose size is in the range of exosomes.

Given the locations of ApoD, would the protein be carried in membrane budding microvesicles (MVs) or in MVB-derived exosomes? When the EV-free medium used to culture cells and the 1321N1-CM collected after 72 h are subjected to NTA (Figure 4D), we found that 1321N1 cells secrete several EV populations, absent in the starting culture media, with a group showing a particle size compatible with exosomes (arrow in Figure 4D, around 100 nm). Following sucrose gradient fractionation of two independent 1321N1 EV preparations and immunoblot analysis, we uncover that ApoD exclusively partitions in the fractions that contain the known density range for exosomes (1.13–1.20 g/ml; (Salomon et al., 2013; Kharaziha et al., 2015). In the preparation shown (Figure 4E), fractions with densities of 1.176 g/ml and 1.232 g/ml are both positive for ApoD and for the exosome marker CD81 (Figure 4E). All fractions are negative for other sub-cellular compartment markers (endoplasmic reticulum chaperone BiP; Figure 4E). Curiously, the ApoD-containing vesicular fractions present both monomeric and dimeric forms of ApoD (see “Discussion” section). Our results demonstrate that ApoD is specifically enriched in the exosome-containing fractions of 1321N1 astroglial EVs.

### ApoD-Containing Exosomes Underlie a Protective Reaction of Glial Cells Against Oxidative Stress and Mediate ApoD-Dependent Neuroprotection

In order to assess whether the presence of ApoD in astrocytic EVs has functional consequences, we tested EV-associated ApoD effect on neuronal viability upon an oxidative insult triggered by the reactive oxygen species (ROS) generator PQ. To address this question we cultured again SH-SY5Y neurons with: (1) EV-free standard media (SM); (2) 1321N1-conditioned media (CM); (3) the EV fraction (EVs) obtained by differential centrifugation of CM; and (4) the EV-depleted supernatant (Sup) of CM.

**Figure 5:**
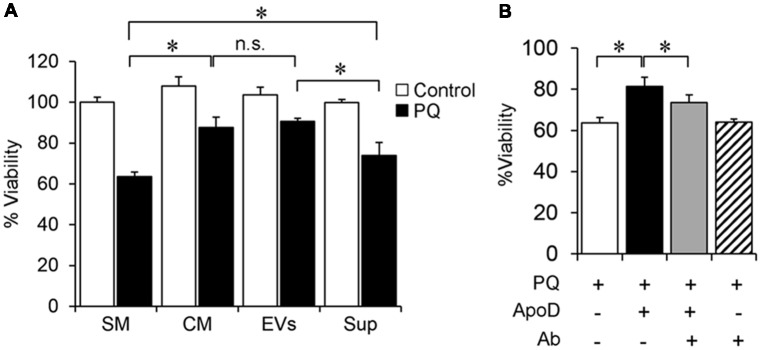
Astroglial EVs promote neuronal survival. **(A)** SH-SY5Y neurons viability in control and oxidative stress (OS) conditions [2 mM paraquat (PQ) for 2 h], measured by the 3-(4,5-dimethylthiazol-2-yl)-2, 5-diphenyltetrazolium bromide (MTT) assay after culture in SM, astrocyte-CM, purified astrocyte EVs, or EV-depleted astrocyte-derived supernatant, Sup.** (B)** Cell viability of PQ-challenged SH-SY5Y neurons (2 mM PQ for 2 h) in the absence or presence of purified native ApoD and/or an equimolar amount of anti-ApoD antibody. Error bars represent SEM (*n* = 3 independent experiments with three technical replicas each). Asterisks represent significant differences (*p* < 0.01) assessed by ANOVA with Holm-Sidak *post hoc* method. (n.s.: non-significant differences) Only the most relevant comparisons are pointed.

In the absence of PQ, the exposure of SH-SY5Y neurons to 1321N1 CM or its EVs does not affect their viability (white bars in Figure 5A). On the other hand, upon exposure to 2 mM PQ for 2 h (black bars in Figure 5A), the decrease observed in SH-SY5Y viability after the OS stimulus is significantly ameliorated after treatment with complete CM or its EV fraction, compared to exposure to PQ in standard medium (SM). Such protection against OS is only partial when the EV-depleted media is used (Sup, black bar, in Figure 5A). Since we have demonstrated that ApoD is uptaken by neurons only in EV format (Figure 3), these results indicate that a full neuroprotection effect by the 1321N1 secretome can only be achieved when EVs are present in the media. The beneficial effect of EV-associated ApoD is comparable to that obtained with native ApoD purified from human cystic fluid (Figure 5B), an effect whose specificity is proven by its reduction when blocking ApoD with a specific antibody.

In summary, our data demonstrate that a fraction of the ApoD produced by 1321N1 astrocytes is targeted to EVs, and particularly to the fraction displaying exosomal properties. We also show that, surprisingly, it is in this form of cell-cell communication, and not in free soluble form, that ApoD gets internalized by SH-SY5Y neurons, where it is able to protect them from PQ-triggered OS.

A fair amount of data account for the neuroprotective effects of ApoD also on astrocytes themselves (Bajo-Grañeras et al., 2011; Pascua-Maestro et al., 2017). To further contrast this hypothesis and expand the finding to non-immortalized native cells, we used the WT and ApoD-KO mice as experimental model.

**Figure 6:**
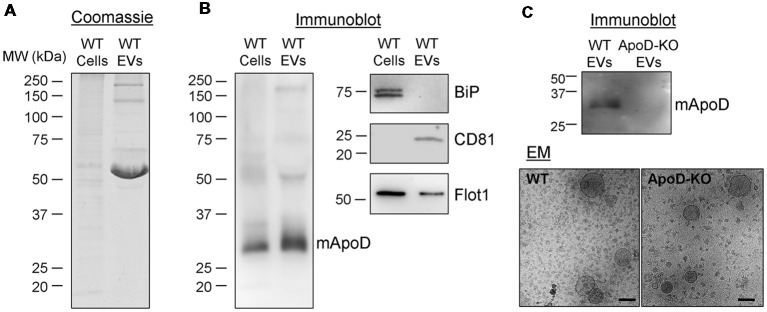
Primary murine astrocytes produce ApoD-positive EVs. **(A)** Coomassie blue staining of whole cell extracts and EV preparation (240 times concentrated, relative to the starting cell culture) from wild-type (WT) primary astrocytes. **(B)** Immunoblot analysis of ApoD, the endoplasmic reticulum marker BiP, and the EV markers CD81 and flotillin-1 in cell lysates and EV preparations from WT primary astrocytes. No signal of BiP is detected in the EV preparation. Intracellular CD81 is not detectable in the cell extract. **(C)** Immunoblot analysis of ApoD in WT and ApoD-knock-out (KO) primary astrocyte EV preparations, confirming the presence of ApoD in WT EVs. Immunoelectron microscopy images of WT and ApoD-KO EVs. Calibration bars: 100 nm.

First, we performed two additional EV purifications from the culture medium of primary murine cortical astrocytes. Immunoblot analysis (Figure 6) demonstrates the presence of ApoD in both the cell homogenate and the EV-enriched fraction from WT primary astrocytes (Figure 6B), but not in the ApoD-KO EV preparations (Figure 6C), after confirming by EM the presence of EVs in the preparations from both genotypes. As expected, EVs show the markers CD81 and flotillin-1 (also in the whole cell extract) while other subcellular compartment marker (the endoplasmic reticulum chaperon BiP) is absent. Taking into account the starting material for the cell homogenate and the EV-enriched preparations from WT astrocytes (Figure 6A), and the relative abundance of ApoD compared with flotillin-1 (Figure 6B), we estimate that a significant proportion (around 0.6%) of the ApoD expressed by an astroglial cell is targeted to EVs.

After demonstrating the secretion of ApoD-loaded EVs by primary WT astrocytes, we tested the effect of astrocyte-derived conditioned media on primary astrocytes of WT and ApoD-KO mice exposed to PQ (500 μM, 2 h).

**Figure 7:**
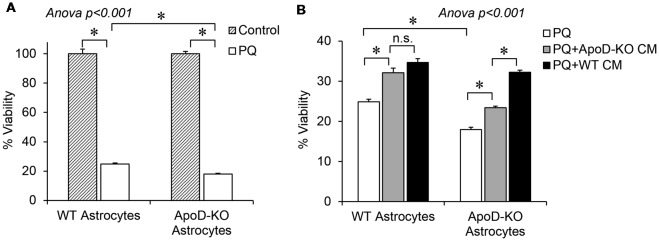
Extracellular ApoD mediates autocrine protection of primary murine astrocytes. **(A)** Percent viability estimated by the MTT assay of WT and ApoD-KO primary astrocytes cultured in SM, in either control or upon an OS challenge with PQ (500 μM, 2 h). ApoD-KO astrocytes are significantly more vulnerable to PQ than WT astrocytes. **(B)** PQ challenge was performed either on SM (white bars), or in astrocyte-CM, either derived from ApoD-KO astrocytes (gray bars) or from WT astrocytes (black bars). Effect of CM on ApoD-KO astrocyte viability is dependent on the genotype of CM origin. Error bars represent SEM (*n* = 3 independent experiments with three technical replicas each). Asterisks point to significant differences (*p* < 0.01) assessed by ANOVA with Holm-Sidak *post hoc* method (n.s.: non-significant differences). Only the most relevant comparisons are pointed.

As expected, ApoD-KO astrocytes showed significantly lower viability than WT astrocytes upon exposure to PQ (Figure 7A). In both cases, the viability significantly improves after culture with astrocyte-derived conditioned media (collected over a 72 h period), regardless of genotype of the cell originating the conditioned media (Figure 7B, gray and black bars). For WT astrocytes, a similar protection effect is attained with media conditioned by either WT or by ApoD-KO cells, which can be explained by the accumulation of various protective factors over time. However, the more vulnerable ApoD-KO astrocytes are significantly better protected when exposed to WT conditioned media (black bar) than when media was conditioned by ApoD-KO astrocytes (gray bar). These results suggest that ApoD, produced by WT astrocytes and present in the extracellular medium, is significantly contributing to protect astrocytes from OS in an autocrine manner.

Together, our results demonstrate that ApoD is present in EVs purified from conditioned media produced by both a human astrocytic cell line and primary murine astrocytes (Figures 4, 6), that ApoD is detected specifically in the exosome-containing fractions of these EVs (Figure 4), and that these ApoD-positive EVs become internalized by SH-SY5Y neurons (Figures 2, 3). Moreover, an unexpected result is the absence of detectable ApoD endocytosis into neurons when we use the EV-depleted CM that strongly indicates that the majority of extracellular ApoD produced by 1321N1 astrocytes is associated to EVs and not in a free soluble form, as previously thought. EV-associated ApoD protects both neurons and astrocytes from PQ-induced OS. These results set the stage to study whether we find *in vivo* such an important contribution of exosomes to the ApoD astrocyte-to-neuron traffic both in physiological and pathological situations.

## Discussion

Besides a role in cell-cell communication during cellular homeostasis, EVs are shed by cells in response to pathological states (reviewed by Croese and Furlan, 2018; Holm et al., 2018). In the nervous system, glial EVs have been involved both in regulation of neuroinflammation (Dickens et al., 2017; Kumar et al., 2017; Li et al., 2018), and in mechanisms triggering neuroprotection (Lopez-Verrilli et al., 2013; Guitart et al., 2016).

The production of ROS and a concomitant inflammatory response have been classically considered a negative side-effect of tissue damage that hampers nervous system recovery upon aging and neurodegeneration. Consequently, great efforts have been made to identify antioxidants that can mediate neuroprotection through improvement of neuronal survival and axonal regeneration. However, a recent report has demonstrated that ROS can also play a positive pro-regenerative role upon neural damage by a mechanism based on glia-neuron EV-mediated Nox2-PI3K–pAkt signaling (Hervera et al., 2018). How can a cell control the side effects of ROS, and the levels that can be managed without tilting the equilibrium towards cell-death?

The Lipocalin ApoD has been demonstrated to be a neuroprotectant by controlling the levels of lipid peroxides generated by ROS accumulation with aging or pathological conditions (Ganfornina et al., 2008; Li et al., 2015; Sanchez et al., 2015; Pascua-Maestro et al., 2017). The neuroprotection exerted by ApoD not only influences ApoD-expressing cells such as astrocytes and myelinating glia, but also affects neurons in a paracrine manner (Bajo-Grañeras et al., 2011; Pascua-Maestro et al., 2017). The presence of a signal peptide in the unprocessed protein and the experimentally verified presence of the mature protein in organelles of the canonical secretion pathway and in the extracellular milieu (Pascua-Maestro et al., 2017), result in the annotation of ApoD as a secreted protein (UniProtKB-P51910/P05090). This property was assumed in the interpretation of our previous studies reporting ApoD traffic in astrocytes and neurons under control or OS conditions (Pascua-Maestro et al., 2017).

The results presented in this work link ApoD traffic to the EV compartment of astrocytes, where it appears very specifically in the exosomal subtype of EVs, as determined by the co-expression of classical markers such as CD81 (Tkach et al., 2018). More importantly, the neuroprotective effect that ApoD exerts upon OS-challenged neurons or astrocytes must be entirely based on the protein present in the EVs supplied by reactive astrocytes, since no neuronal ApoD expression is triggered by the EVs, and no incorporation of astrocyte-derived ApoD is detected when EVs have been removed from the astrocyte-CM.

This result agrees with other reports showing that non-neural cells exposed to ROS release EVs that carry OS response proteins and antioxidants (Saeed-Zidane et al., 2017; Chettimada et al., 2018). The fact that EV-associated human ApoD is detected both as monomers and dimers (Figure 4), is a readout of its antioxidant activity, since it is known that a consequence of its lipid reducing activity is the formation of stable dimers (Bhatia et al., 2012) that accumulate in advanced stages of Alzheimer’s disease patients (Bhatia et al., 2013). Since we know that the1321N1 astrocytic cell line has a basal level of OS (Pascua-Maestro et al., 2017), it is not surprising that they are already targeting the redox pair reduced/oxidized-ApoD to exosomes.

The discovery of ApoD in glia-derived EVs, and particularly in exosome-like fractions, reframes our understanding of the neuroprotective role of this Lipocalin. The capability of EVs to cross the blood-brain barrier (Krämer-Albers, 2017) opens up new research avenues to explore the use of systemically administered ApoD-positive exosomes to treat neurodegenerative diseases.

## Author Contributions

RP-M, MG, JF-P and DS conceived and designed the project. RP-M, EG, CL and MG designed and performed the experiments. RP, EG, JF-P and DS analyzed the data. DS, MG and JF-P supervised the project. RP-M, EG, CL, JF-P, MG and DS wrote the article.

## Conflict of Interest Statement

The authors declare that the research was conducted in the absence of any commercial or financial relationships that could be construed as a potential conflict of interest.
